# BRCA1: A Novel Prognostic Factor in Resected Non-Small-Cell Lung Cancer

**DOI:** 10.1371/journal.pone.0001129

**Published:** 2007-11-07

**Authors:** Rafael Rosell, Marcin Skrzypski, Ewa Jassem, Miquel Taron, Roberta Bartolucci, Jose Javier Sanchez, Pedro Mendez, Imane Chaib, Laia Perez-Roca, Amelia Szymanowska, Witold Rzyman, Francesco Puma, Grazyna Kobierska-Gulida, Raffaele Farabi, Jacek Jassem

**Affiliations:** 1 Catalan Institute of Oncology, Hospital Germans Trias i Pujol, Badalona, Spain; 2 Medical University of Gdansk, Gdansk, Poland; 3 Azienda Ospedaliera Santa Maria, Terni, Italy; 4 Autonomous University of Madrid, Madrid, Spain; Memorial Sloan-Kettering Cancer Center, United States of America

## Abstract

**Background:**

Although early-stage non-small-cell lung cancer (NSCLC) is considered a potentially curable disease following complete resection, patients have a wide spectrum of survival according to stage (IB, II, IIIA). Within each stage, gene expression profiles can identify patients with a higher risk of recurrence. We hypothesized that altered mRNA expression in nine genes could help to predict disease outcome: excision repair cross-complementing 1 (ERCC1), myeloid zinc finger 1 (MZF1) and Twist1 (which regulate N-cadherin expression), ribonucleotide reductase subunit M1 (RRM1), thioredoxin-1 (TRX1), tyrosyl-DNA phosphodiesterase (Tdp1), nuclear factor of activated T cells (NFAT), BRCA1, and the human homolog of yeast budding uninhibited by benzimidazole (BubR1).

**Methodology and Principal Findings:**

We performed real-time quantitative polymerase chain reaction (RT-QPCR) in frozen lung cancer tissue specimens from 126 chemonaive NSCLC patients who had undergone surgical resection and evaluated the association between gene expression levels and survival. For validation, we used paraffin-embedded specimens from 58 other NSCLC patients. A strong inter-gene correlation was observed between expression levels of all genes except NFAT. A Cox proportional hazards model indicated that along with disease stage, BRCA1 mRNA expression significantly correlated with overall survival (hazard ratio [HR], 1.98 [95% confidence interval (CI), 1.11-6]; P = 0.02). In the independent cohort of 58 patients, BRCA1 mRNA expression also significantly correlated with survival (HR, 2.4 [95%CI, 1.01-5.92]; P = 0.04).

**Conclusions:**

Overexpression of BRCA1 mRNA was strongly associated with poor survival in NSCLC patients, and the validation of this finding in an independent data set further strengthened this association. Since BRCA1 mRNA expression has previously been linked to differential sensitivity to cisplatin and antimicrotubule drugs, BRCA1 mRNA expression may provide additional information for customizing adjuvant antimicrotubule-based chemotherapy, especially in stage IB, where the role of adjuvant chemotherapy has not been clearly demonstrated.

## Introduction

In 2006 in Europe, there were an estimated 386,300 lung cancer cases, with a substantially higher incidence in men than in women[1]. Among completely resected non-small-cell lung cancer (NSCLC) patients, 40% of stage I, 66% of stage II and 75% of stage IIIA patients die within five years of resection[2], and the benefit of adjuvant chemotherapy has not been demonstrated in stage IB. In the ANITA randomized trial, 5-year survival for patients with stage IB disease was 62% in the chemotherapy group and 64% in the control group; corresponding rates were 52% and 39% for stage II patients and 42% and 26% for stage IIIA[3]. In addition to disease stage, several studies have examined gene expression profiles in NSCLC, identifying molecular subtypes associated with patient outcome[4], [5], [6], [7]. Gene expression signatures ranging from five to 64 genes have been identified[6], [7], and cross-study comparisons have revealed significant, though incomplete, agreement of patterns predicting outcome[4]. Moreover, the ability to interpret the meaning of the individual genes in these signatures remains a challenge[8]. The gene expression signatures identified genes mostly related to cancer metastasis[6] but did not describe genes involved in DNA repair pathways.

Preclinical studies have demonstrated that a deficiency in any one of the more than 30 genes involved in the nucleotide excision repair (NER) pathway confers marked hypersensitivity to cisplatin[9]. Hypothesizing that elevated levels of NER genes could be not only predictive but also prognostic markers, we chose to examine the following genes, based on previous reports of their predictive value: excision repair cross-complementing 1 (ERCC1)[10], BRCA1[11], human homolog of yeast budding uninhibited by benzimidazole (BubR1)[12], [13], [14], myeloid zinc finger 1 (MZF1)[15], [16], [17], ribonucleotide reductase subunit M1 (RRM1)[18], [19], [20], thioredoxin-1 (TRX1)[21], [22], tyrosyl-DNA phosphodiesterase (Tdp1)[23], [24], [25]. In addition, we examined Twist[26], [27] and nuclear factor of activated T cells (NFAT)[28], [29], which are involved in the invasion-metastasis process. (Further details on the nine genes examined can be found in Text S1.)

None of these nine genes have been identified in gene expression profiles associated with NSCLC patient outcome[4], [5], [6], [7], with the exception of TRX1, which was identified by proteomic analysis and associated with poor survival[21]. In order to shed light on the prognostic value of these genes, we have examined their expression by real-time quantitative reverse transcriptase PCR (RT-PCR) in 126 completely resected NSCLC patients who did not receive adjuvant chemotherapy and correlated the results with survival.

## Methods

### Patients

NSCLC samples were obtained from 126 consecutive patients who underwent curative pulmonary resection at the Medical University of Gdansk (Gdansk, Poland) between 2000 and 2004, after obtaining approval from the institutional review board of the Medical University of Gdansk and patients' signed informed consent. The patients were 98 males and 28 females, with age at diagnosis ranging from 37 to 77 years (median age, 64 years). Seventy-one patients had stage I disease, 33 stage II, and 22 stage IIIA. Twenty-seven patients had poorly differentiated, 74 moderately differentiated, and 9 well-differentiated NSCLC; the remaining 16 patients were unspecified. Eighty patients were smokers, 39 former smokers, and the remaining seven never-smokers. One hundred and twenty-two patients underwent formal pulmonary lobectomy or more, with systematic ipsilateral mediastinal lymph node dissection; the four remaining patients underwent segmentectomy due to poor pulmonary reserve. Stages were determined after pathologic evaluation of resected specimens according to the International System for Staging Lung Cancer[30] (Table S1). None of the patients received adjuvant chemotherapy.

We validated the BRCA1 prognostic value in 58 stage IB-IIB NSCLC patients who had undergone surgical resection at the Azienda Ospedaliera Santa Maria (Terni, Italy) between February 1997 and December 2003, after obtaining approval from the institutional review board of Azienda Ospedaliera Santa Maria and patients' signed informed consent. Patient characteristics are shown in Table S1.

### Gene expression analysis

Tumor samples from the 126 patients were obtained during surgery as blocks of 1cm3 and snap-frozen in liquid nitrogen. Tissues were stored in −80°C until total RNA was extracted with AllPrep kits (Qiagen, Valencia, CA). Only tumor samples containing more than 60% of tumor tissue on a microscopic section were eligible for further processing. The concentration of RNA was assessed in Nano-drop™ and the quality of obtained RNA was tested on agarose gel. First-strand cDNA was synthesized from 1 µg of total RNA using the High-Capacity cDNA Archive Kit (Applied Biosystems, Foster City, CA). The nine genes examined are shown in Table 1. Quantitative RT-PCR reactions of each gene were done in an ABI PRISM 7900 HT Sequence Detection System (Applied Biosystems).

**Table 1:** Nine genes examined in this study

Official Symbol	Name	Accession number (transcript) *(GEN ID)*	Aliases
ERCC1	excision repair cross-complementing rodent repair deficiency, complementation group 1	NM_001983	COFS4, UV20
		NM_202001	
		*(2067)*	
MZF1	myeloid zinc finger 1	NM_198055	MZF-1, MZF1B, ZNF42, ZSCAN6, Zfp98
		NM_003422	
		*(7593)*	
Twist1	twist homolog 1 (acrocephalosyndactyly 3; Saethre-Chotzen syndrome) (Drosophila)	NM_000474.3	ACS3, BPES2, BPES3, SCS, TWIST
		*(7291)*	
RRM1	ribonucleotide reductase M1 polypeptide	NM_001033	R1, RIR1, RR1
		*(6240)*	
TXN	thioredoxin	NM_003329	DKFZp686B1993, MGC61975, TRX
		*(7295)*	
Tdp1	tyrosyl-DNA phosphodiesterase	NM_018319	FLJ11090, MGC104252
		NM_001008744	
		*(55775)*	
NFATC2	nuclear factor of activated T-cells, cytoplasmic, calcineurin-dependent 2	NM_173091	RP5-1009H6.1, KIAA0611, NFAT1, NFATP
		NM_012340	
		*(4773)*	
BRCA1	breast cancer 1, early onset	NM_007294	BRCAI, BRCC1, IRIS, PSCP, RNF53
		*(672)*	
BUB1B	BUB1 budding uninhibited by benzimidazoles 1 homolog beta (yeast)	NM_001211	BUB1beta, BUBR1, Bub1A, MAD3L, SSK1, hBUBR1
		*(701)*	

Relative gene expression values were calculated by the ΔΔCt method using the Sequence Detection System (SDS) 2.1 software (Applied Biosystems). The ΔΔCt method gives the amount of target gene normalized to an endogenous reference gene (ribosomal 18S RNA) and relative to a calibrator sample (reference for all samples; commercially available Normal Lung and Liver Human RNA (Stratagene, La Jolla, CA). Primers for the nine genes are listed in Table S2.

ERCC1, RRM1 and BRCA1 gene expression was assessed in formalin-fixed, paraffin-embedded surgical specimens from the 58 patients in the validation cohort. Using laser capture microdissection technique (Palm Microlaser, Oberlensheim, Germany) ensured a minimum of 80% of tumor tissue. After standard tissue sample deparaffinization using xylene and alcohols, samples were lysed in a tris-chloride, EDTA, sodium dodecyl sulphate (SDS) and proteinase K containing buffer. RNA was then extracted with phenol-chloroform-isoamyl alcohol followed by precipitation with isopropanol in the presence of glycogen and sodium acetate. RNA was resuspended in DEPC water (Ambion Inc, Austin TX, USA) and treated with DNAse I (Ambion Inc) to avoid DNA contamination. cDNA was synthesized using M-MLV retrotranscriptase enzyme. Template cDNA was added to Taqman Universal Master Mix (Applied Biosystems) in a 12.5-μl reaction with specific primers and probe for each gene. The primer and probe sets were identical to those used in the frozen specimens; the endogenous reference gene was β-actin. Quantification of gene expression was performed using the ABI Prism 7900HT Sequence Detection System (Applied Biosystems).

### Statistical analyses

Median values and ranges were derived for quantitative variables and mRNA gene expression. Qualitative variables were summarized by means of absolute frequencies and percentages. The Kruskal-Wallis test was used to check for normality. Differences in median mRNA expression levels between histological types were assessed by the U Mann-Whitney test. Spearman's rank-correlation coefficient (rho) was used to measure the correlations among gene expression levels. We made an *a priori* decision to classify mRNA gene-expression levels as high or low, using the minimum P value method modified by Lausen and Schumacher[31]. The Bonferroni method was used for the correction of the effect of multiple comparisons, and empirical P values for each gene were confirmed through 5000 permutation tests. When no optimal cut-off point was found, we used the sample median for the analysis of time to relapse and survival. Time to relapse and survival were calculated using Kaplan–Meier estimates and differences between curves were tested using the log-rank test. To choose an appropriate subset of genes for association to any clinical variable (histology, stage and grade), we performed a forward and backward Cox regression analysis. For all calculations, the tests performed were two-sided, significance was set at 5%, and the power was 80%. Analyses were performed using Statistical Package for the Social Sciences (SPSS) for Windows version 14 (SPSS Inc, Chicago, IL) and S-Plus 6.1 for Windows.

## Results

The median values of each gene for the entire group of samples are shown in Table S3. Gene amplification was not successful in a minority of samples for every transcript analyzed. There were significant differences in expression according to histology for all genes except NFAT, with higher levels observed in squamous cell carcinomas than in adenocarcinomas (Table 2). There were no differences in gene expression according to stage (Table S4). A strong correlation was observed between expression levels of different genes, for example, between levels of TRX and RRM1 (rho = 0.52; P = 0.0003) and between ERCC1 and BRCA1 (rho = 0.62; P = 0.0001) (Table 3).

**Table 2:** Gene expression according to histology

	Squamous cell carcinoma	Adenocarcinoma	P*
	Median (range)	Median (range)	
ERCC1	1.41 (0.45–7.34)	0.72 (0.23–2.45)	0.0001
MZF1	0.62 (0.06–6.72)	0.25 (0.03–1.49)	0.0001
Twist	10.37 (0.30–76.01)	2.50 (0.14–19.16)	0.0001
RRM1	2 (0.6–6.9)	1.2 (0.4–2.9)	0.0001
TRX	2.13 (0.40–11.88)	0.91 (0.31–7.94)	0.0001
Tdp1	1.7 (0.6–7.3)	1.3 (0.1–2.6)	0.02
NFAT	0.4 (0.1–2.3)	0.5 (0.1–1.8)	0.65
BRCA1	4.26 (0.55–18.48)	1.50 (0.09–8.08)	0.0001
BubR1	16.3 (1.4–90)	7 (0.8–25)	0.0001

* Mann-Whitney U

**Table 3:** Correlation between expression levels of the nine genes examined

	ERCC1	MZF1	Twist	RRM1	TRX	Tdp1	NFAT	BRCA1
MZF1	0.70							
	P = 0.0001							
Twist	0.55	0.29						
	P = 0.0001	P = 0.001						
RRM1	0.33	0.27	0.24					
	P = 0.0001	P = 0.004	P = 0.01					
TRX	0.39	0.20	0.25	0.52				
	P = 0.0001	P = 0.03	P = 0.005	P = 0.0001				
Tdp1	0.25	0.25	0.10	0.65	0.34			
	P = 0.007	P = 0.008	P = 0.26	P = 0.0001	P = 0.0001			
NFAT	0.18	0.41	−0.09	−0.19	−0.01	−0.04		
	P = 0.06	P = 0.0001	P = 0.36	P = 0.04	P = 0.88	P = 0.66		
BRCA1	0.62	0.58	0.29	0.62	0.48	0.47	0.04	
	P = 0.0001	P = 0.0001	P = 0.001	P = 0.0001	P = 0.0001	P = 0.0001	P = 0.69	
BubR1	0.12	0.30	0.23	0.83	0.47	0.63	−0.17	0.69
	P = 0.0001	P = 0.001	P = 0.01	P = 0.0001	P = 0.0001	P = 0.0001	P = 0.06	P = 0.0001

**Figure 1:**
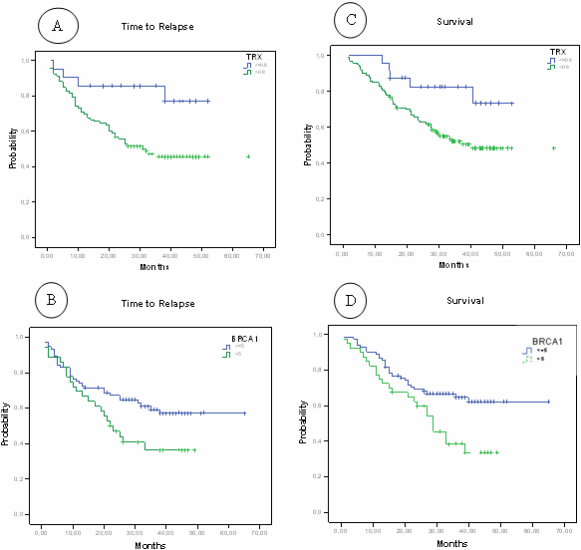
Event-free (A,B) and median (C,D) survival according to expression of TRX (A,C) and BRCA1 (B,D).

With a median follow-up of 29.7 months (range, 1.7–65.9 months), overall event-free and median survival have not been reached. When event-free and median survival was analyzed according to expression levels of the nine genes, TRX and BRCA1 showed significant differences. Event-free survival for 21 patients with low TRX levels has not been reached, while it was 32 months (95%CI, ) for the remaining 93 patients with high levels (P = 0.02). For 77 patients with low levels of BRCA1, event-free survival has not been reached, while it was 22 months (95%CI, 14.9–29 months) for those with high levels (P = 0.04) (Table S5, Fig. 1). Event-free survival curves according to expression of the other seven genes are shown in Figure S1. Median survival for 24 patients with low TRX levels has not been reached, while it was 39 months for the remaining 101 patients with high levels (P = 0.03). For 83 patients with low levels of BRCA1, median survival has not been reached, while it was 29 months (95%CI, 22.2–35.7 months) for those with high levels (P = 0.04) (Table 4, Fig. 1). Median survival curves according to expression of the other seven genes are shown in Figure S2. However, when only stage I patients were examined, event-free survival was significantly different according to expression levels of MZF1 and BRCA1 (Table S6, Fig. S3), and median survival was significantly different according to expression levels of ERCC1, MZF1, Twist and BRCA1 (Table S7, Fig. S4).

**Table 4:** Median survival according to gene expression

	N*	Median Survival (months)	95% CI	P
ERCC1				0.89
≤1.24	61	NR	-	
>1.24	62	39.5	-	
MZF1				0.05
≤0.5	56	NR	-	
>0.5	66	33	21.9–44.1	
Twist				0.39
≤7.75	61	NR	-	
>7.75	61	NR	-	
RRM1				0.11
≤1.65	61	NR	-	
>1.65	60	33.9	24–43.9	
TRX				0.03
≤0.8	24	NR	-	
>0.8	101	39	-	
Tdp1				0.88
≤1.57	60	NR	-	
>1.57	61	41.3	-	
NFAT				0.51
≤0.46	61	NR	-	
>0.46	61	33.9	-	
BRCA1				0.01
≤5	83	NR	-	
>5	40	29	22.2–35.7	
BubR1				0.41
≤12.28	61	NR	-	
>12.28	61	36.7	-	

* Survival data is not available for some patients. Gene amplification was not successfully performed in all samples for all genes.

NR = not reached

The Cox proportional hazards model selected pathological stage IIIA and BRCA1 expression as independent prognostic factors for survival. The hazard ratio (HR) was 7.91 (95%CI, 2.27-27.54; P = 0.001) for stage IIIA and 1.98 (95%CI, 1.11-6); P = 0.02) for BRCA1 expression (Table S8).

### Validation of BRCA1

The median follow-up of the 58 patients in the validation cohort was 40 months. According to the Cox proportional hazards model, the HR for patients with high levels of BRCA1 was 2.4 (95%CI, 1.01-5.92; P = 0.04). There were no stage IIIA patients in this cohort.

## Discussion

Gene expression signatures have been shown to predict outcome in resected stage I NSCLC[5], [6], However, the use of microarrays is limited due to the need for fresh-frozen tissue. RT-QPCR involving a small number of genes offers a practical alternative, allowing for accurate and reproducible quantification of results for RNA obtained from small amounts of paraffin-embedded specimens. The results of RT-QPCR performed on five[7] and eight[32] genes correlated with outcomes of NSCLC[7] and lung adenocarcinoma[32] patients. We have examined the expression of ERCC1, BRCA1, BubR1, MZF1, RRM1, TRX1 and Tdp1, involved in DNA repair pathways, and of Twist and NFAT , related to metastasis formation. In the multivariate model, only BRCA1 and stage IIIA were identified as independent prognostic variables. In an independent validation cohort of 58 stage IB-IIB NSCLC patients, BRCA1 was confirmed as the only independent prognostic marker.

Patients whose tumors had high BRCA1 expression had significantly worse survival and should be candidates for adjuvant chemotherapy. *In vitro* studies have shown that BRCA1 can regulate differential sensitivity to different classes of chemotherapy agents[33]. The absence of BRCA1 results in high sensitivity to cisplatin, whereas its presence increases sensitivity to antimicrotubule agents[33]. Therefore, we believe that patients with the highest expression levels should receive antimicrotubule, non-platinum-based chemotherapy. We have carried out a pilot study of customized adjuvant chemotherapy based on BRCA1 mRNA levels in 88 completely resected stage II-IIIA NSCLC patients, where those with the highest expression levels received adjuvant docetaxel and those with lower levels received cisplatin-based chemotherapy. The interim analysis shows that event-free survival is similar in both groups. These findings support our previous findings in stage II-IIIA patients who received neoadjuvant gemcitabine/cisplatin, where those with the highest BRCA1 levels had a dismal survival of 12 months[11].

No differences in expression levels of any of the nine genes were observed according to stage or tumor size (<4 vs >4 cms). However, of all the nine genes examined, only BRCA1 showed a trend towards influencing survival according to tumor size. In stage I NSCLC patients, survival has been inversely correlated with tumor size[34]. In the present study, the univariate survival analysis showed that in addition to BRCA1, ERCC1 and MZF1 significantly influenced survival in stage I (Table S7, Fig. S4). These findings highlight the potential role of ERCC1 and MZF1, which are highly correlated with BRCA1, as strong prognostic markers in stage I NSCLC. Not unexpectedly, however, considering the high correlation between the expression levels of these three genes (Table 3), when all three genes were combined, no further improvement over the prognostic value of BRCA1 alone was observed.

Although the mechanisms by which some of the nine genes examined affect patient prognosis is not very clear, overexpression of ERCC1 and RRM1 seems to be oncogene-driven[35], [36], [37], [38], [39]. BRCA1 methylation and abrogation of BRCA1 mRNA has been found in sporadic breast cancers[40] but very rarely in NSCLC[41]. In some sporadic breast cancers, the poor outcome associated with BRCA1 methylation and low levels of expression could be explained by MYC amplification[42].

Other studies, using the monoclonal antibody 8F1[43] have reported that the presence of ERCC1 protein is a prognostic marker of survival in early NSCLC and a predictor of outcome to adjuvant cisplatin-based chemotherapy; however, in a prior study in gastric cancer[44], it was unclear whether the poor clinical response of patients whose tumors had high pretreatment mRNA levels of ERCC1 resulted from tumor cell resistance to cisplatin-based chemotherapy or from a more aggressive tumor biology. Moreover, in ERCC1-positive normal human fibroblasts and cells from patients with inherited mutations in ERCC1, ERCC1 is not the principal antigen recognized by the 8F1 antibody on immunostaining[45]. Furthermore, in another study, ERCC1 protein status did not correlate with survival in stage IV NSCLC[46], while in a trial of customized cisplatin based on ERCC1 mRNA expression, response rate was 39% in the control arm and 50% in the customized arm (P = 0.02)[47].

In summary, our study indicates that firstly, BRCA1 is closely related to ERCC1, RRM1 and other genes like MZF1, but stands out as the most significant prognostic marker of relapse. We hypothesize that patients with high BRCA1 levels will benefit from antimicrotubule-based–but not cisplatin-based–chemotherapy. Secondly, high levels of these transcripts confer a higher risk of relapse, in contrast to what has been reported by other investigators, which highlights the need for further research in this area to elucidate the predictive role of these NER-related genes and to correctly customize treatment (Fig. S5). Although the population in our study was skewed to male smokers with squamous cell carcinoma, our results warrant further investigation to confirm their applicability to other histological subsets of NSCLC, In order to shed further light on these issues, we are planning to examine BRCA1, ERCC1, MZF1 and RRM1 expression in 200 tumor specimens from the ANITA study[3] and in 620 patients included in the Spanish Lung Cancer Group NATCH trial of neoadjuvant vs adjuvant chemotherapy vs surgery alone.

## Supporting Information

Figure S1 Event-free survival according to the expression of ERCC1 (A), MZF1 (B), Twist (C), RRM1 (D), Tdp1 (E), NFAT (F), and BubR1 (G)
(0.09 MB TIF)

Figure S2 Median survival according to the expression of ERCC1 (A), MZF1 (B), Twist (C), RRM1 (D), Tdp1 (E), NFAT (F), and BubR1 (G)
(0.09 MB TIF)

Figure S3 Event-free survival curves for stage I patients according to gene expression levels of the nine genes examined
(0.10 MB TIF)

Figure S4 Median survival curves for stage I patients according to gene expression levels of the nine genes examined
(0.10 MB TIF)

Figure S5 Contradictory findings leading to opposed strategies of customizing adjuvant chemotherapy. Olaussen et al (NEJM 2006;355:983-991) report that the lack of ERCC1protein implies a higher risk of relapse and a greater sensitivity to cisplatin-based chemotherapy. (Cisplatin sensitivity based on lack of ERCC1 expression has been demonstrated in preclinical and clinical studies.) Our findings indicate that a higher risk of relapse is related to high levels of several transcripts, including ERCC1. These patients could be resistant to cisplatin and sensitive to taxanes or other antimicrotubule drugs
(0.13 MB TIF)

Table S1 Patient characteristics for principal cohort (N = 126) and for validation cohort (N = 58)
(0.04 MB DOC)

Table S2 Primers and probes for the nine genes examined
(0.03 MB DOC)

Table S3 Relative gene expression values
(0.03 MB DOC)

Table S4 Gene expression according to disease stage
(0.04 MB DOC)

Table S5 Event-free survival according to gene expression levels
(0.06 MB DOC)

Table S6 Event-free survival in stage I patients according to gene expression levels
(0.06 MB DOC)

Table S7 Median survival for stage I patients according to gene expression levels
(0.06 MB DOC)

Table S8 Multivariate Cox model for survival, showing a greater risk of death for patients with high levels of BRCA1 and for those with stage IIIA disease
(0.03 MB DOC)

Text S1 Further details on the nine genes examined
(0.09 MB DOC)
