# Supplementary figures and images for: BRCA1: A Novel Prognostic Factor in Resected Non-Small-Cell Lung Cancer
Source: PLoS One. 2007 Nov 7;2(11):e1129. doi: 10.1371/journal.pone.0001129 (PMC2042516; doi:10.1371/journal.pone.0001129)

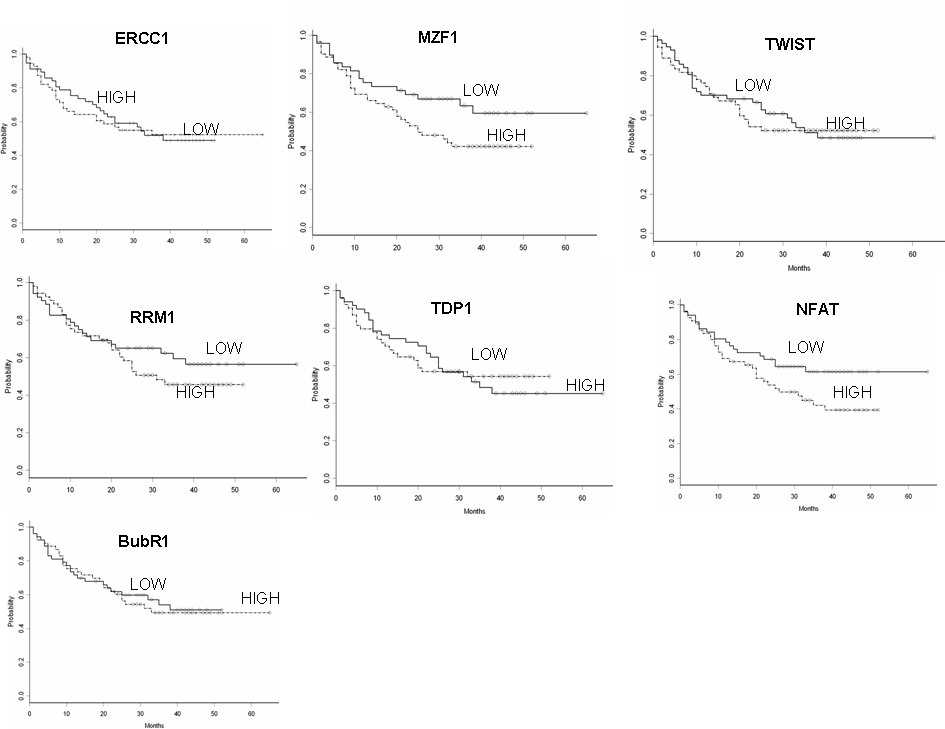

Supplement: Figure S1 — Event-free survival according to the expression of ERCC1 (A), MZF1 (B), Twist (C), RRM1 (D), Tdp1 (E), NFAT (F), and BubR1 (G) (0.09 MB TIF) [file pone.0001129.s001.tif]

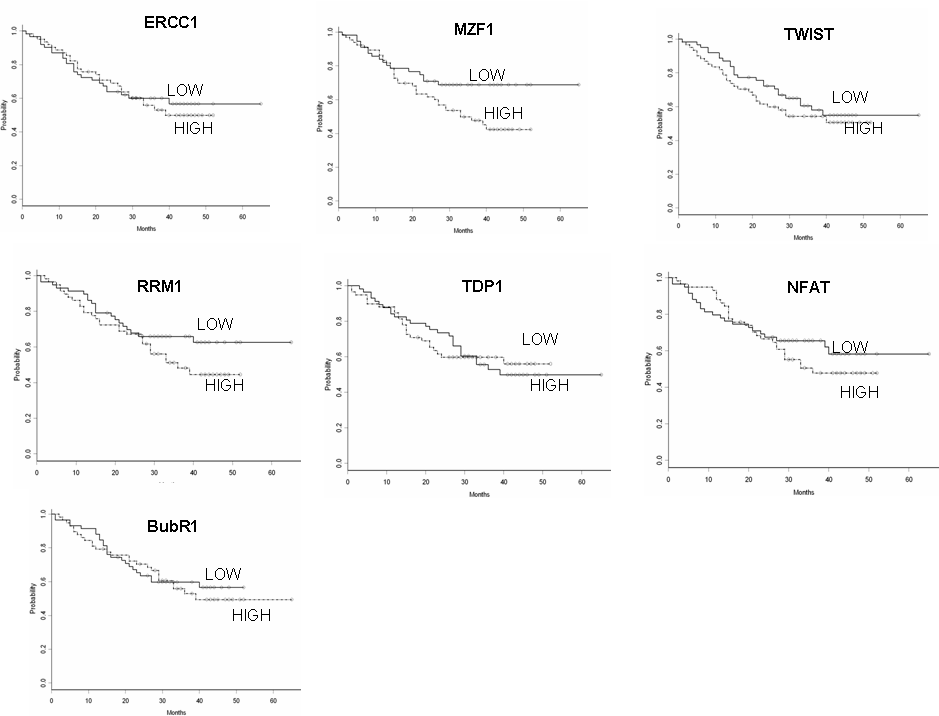

Supplement: Figure S2 — Median survival according to the expression of ERCC1 (A), MZF1 (B), Twist (C), RRM1 (D), Tdp1 (E), NFAT (F), and BubR1 (G) (0.09 MB TIF) [file pone.0001129.s002.tif]

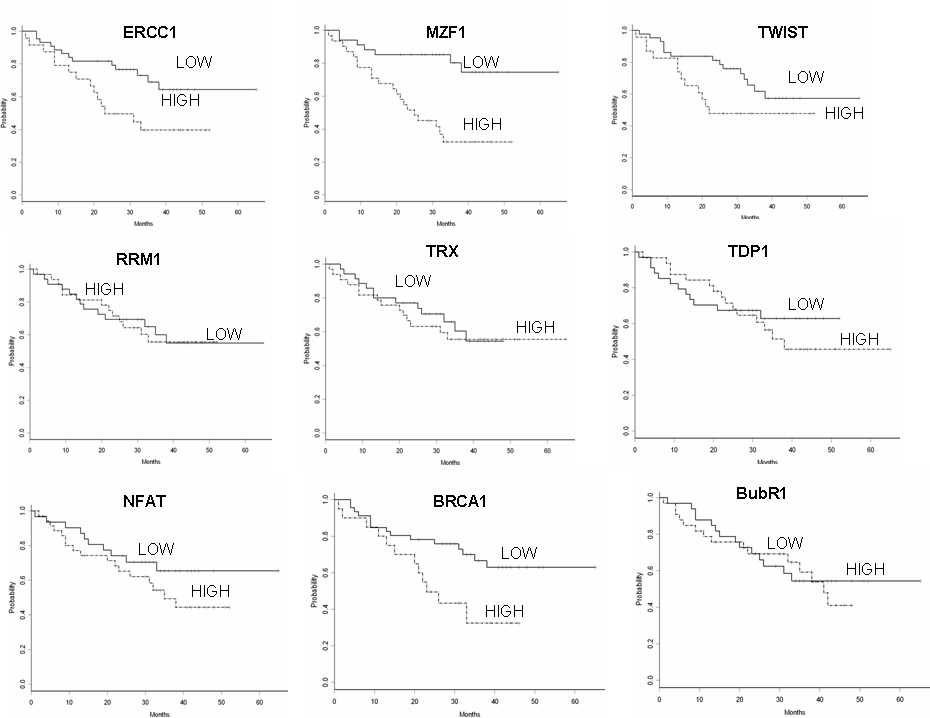

Supplement: Figure S3 — Event-free survival curves for stage I patients according to gene expression levels of the nine genes examined (0.10 MB TIF) [file pone.0001129.s003.tif]

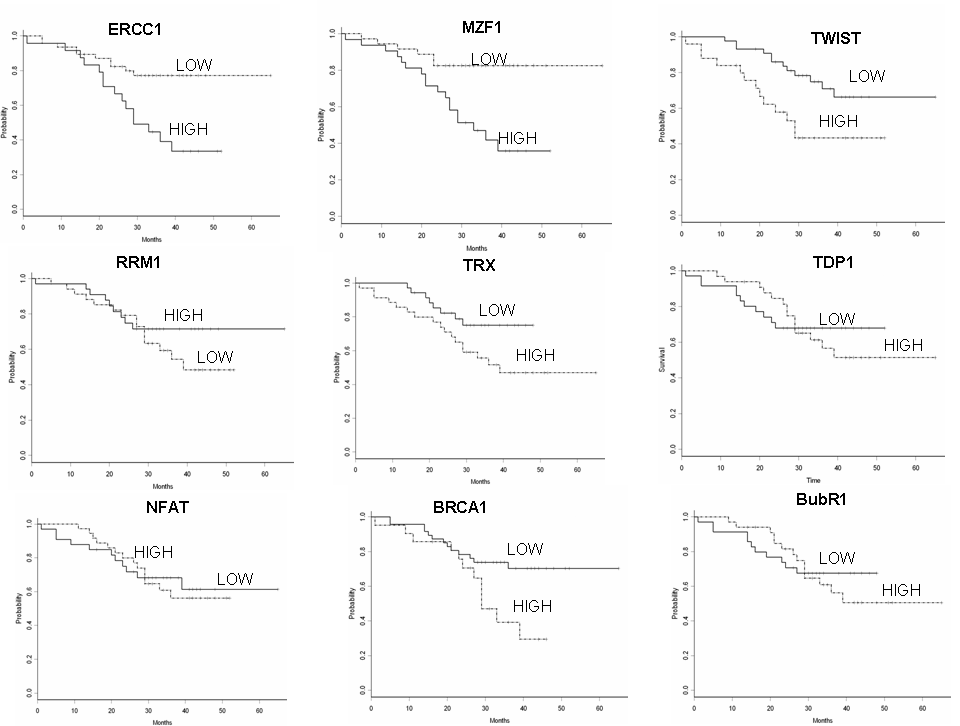

Supplement: Figure S4 — Median survival curves for stage I patients according to gene expression levels of the nine genes examined (0.10 MB TIF) [file pone.0001129.s004.tif]

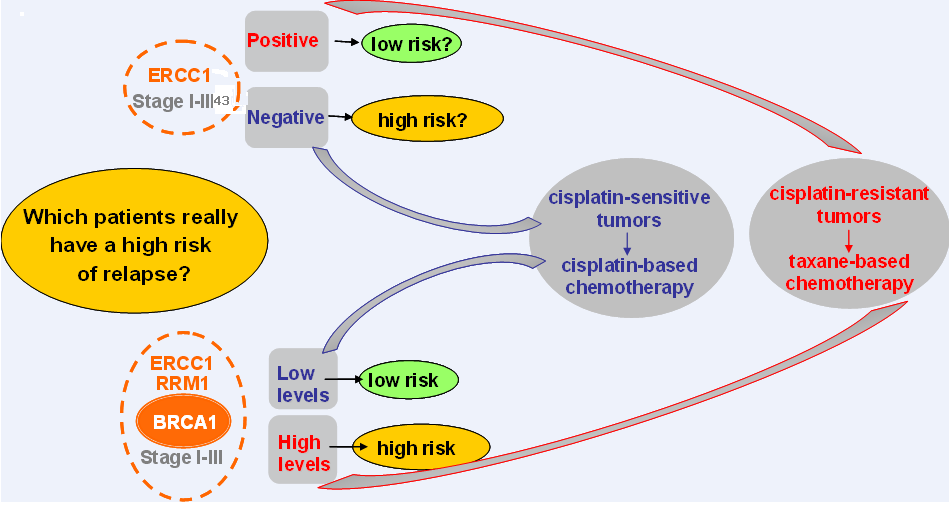

Supplement: Figure S5 — Contradictory findings leading to opposed strategies of customizing adjuvant chemotherapy. Olaussen et al (NEJM 2006;355:983-991) report that the lack of ERCC1protein implies a higher risk of relapse and a greater sensitivity to cisplatin-based chemotherapy. (Cisplatin sensitivity based on lack of ERCC1 expression has been demonstrated in preclinical and clinical studies.) Our findings indicate that a higher risk of relapse is related to high levels of several transcripts, including ERCC1. These patients could be resistant to cisplatin and sensitive to taxanes or other antimicrotubule drugs (0.13 MB TIF) [file pone.0001129.s005.tif]
