# Supplementary material for: BRCA1: A Novel Prognostic Factor in Resected Non-Small-Cell Lung Cancer
Source: PLoS One. 2007 Nov 7;2(11):e1129. doi: 10.1371/journal.pone.0001129 (PMC2042516; doi:10.1371/journal.pone.0001129)
Supplement: Table S1 — Patient characteristics for principal cohort (N = 126) and for validation cohort (N = 58) (0.04 MB DOC) [file pone.0001129.s006.doc]

| **Principal cohort (N=126)** | **N (%)** |
| --- | --- |
| Age median (range) | 64 (37-103) |
| Sex  Female  Male | 28 (22.2)  98 (77.8) |
| Smoking History  Never  Current  Former | 7 (5.5)  80 (63.5)  39 (31) |
| Surgery  Segmentectomy  Lobectomy  Bilobectomy  Pneumonectomy | 4 (3.2)  69 (54.8)  13 (10.3)  40 (31.7) |
| Histology  Squamous Cell Carcinoma  Adenocarcinoma | 93 (73.8)  33 (26.2) |
| Stage  IA  IB  II  IIIA | 18 (14.3)  53 (42.1)  33 (26.2)  22 (17.5) |
| Grade  Well-differentiated  Moderately differentiated  Poorly differentiated  Unspecified | 9 (8.2)  74 (67.3)  27 (24.5)  16 |
|  |  |
| **Validation cohort (N=58)** | **N (%)** |
| Age median (range) | 67.5 (48-85) |
| Sex  Female  Male | 3 (5.2)  55 (94.8) |
| Smoking History  Never  Current | 5 (8.6)  53 (91.4) |
| Surgery  Lobectomy  Bilobectomy  Pneumonectomy | 43 (74.1)  6 (10.3)  9 (15.5) |
| Histology  Squamous Cell Carcinoma  Adenocarcinoma | 45 (77.6)  13 (22.4) |
| Stage  IB  IIA  IIB | 37 (63.8)  7 (12.1)  14 (24.1) |
