# Supplementary material for: BRCA1: A Novel Prognostic Factor in Resected Non-Small-Cell Lung Cancer
Source: PLoS One. 2007 Nov 7;2(11):e1129. doi: 10.1371/journal.pone.0001129 (PMC2042516; doi:10.1371/journal.pone.0001129)
Supplement: Table S2 — Primers and probes for the nine genes examined (0.03 MB DOC) [file pone.0001129.s007.doc]

| **Gene** | **Forward Primer (5’-3’)** | **Reverse Primer (5’-3’)** | **Probe (5’-3’)** |
| --- | --- | --- | --- |
| ERCC1 | GGG AAT TTG GCG ACG TAA TTC | GCG GAG GCT GAG GAA CAG | CAC AGG TGC TCT GGC CCA GCA CAT A |
| MZF1 | AGAGGTTACAGAGGACTCAGATTTCC | GGCAGGAGGGTGGGTACAG | TGGGCCTCTAGCTGC |
| Twist1 | Assay from Applied Biosystems nº Hs_00361186_m1 | | |
| RRM1 | ACT AAG CAC CCT GAC TAT GCT ATC C | CTT CCA TCA CAT CAC TGA ACA CTT T | CAG CCA GGA TCG CTG TCT CTA ACT TGC A |
| TXN | TCCAACGTGATATTCCTTGAAGTAGA | CATGCATTTGACTTCACACTCTGA | ATGACTGTCAGGATGTTG |
| Tdp1 | TATCTCCTTTATTTGGGACGCTTG | GCCAGTCCACGTCAAAGCA 3’ | CTTCAGCTCAGTTTAACTAC |
| NFATC2 | CCCTTGGAGCCCAAAAACA | CGGCGTTTCTAAGCTTCAAGA | GGGCAACCATCGACT |
| BRCA1 | GGCTATCCTCTCAGAGTGACATTTTA | GCTTTATCAGGTTATGTTGCATGGT | CCACTCAGCAGAGGG |
| BUB1B | TGGCGGCGGTGAAGAA | TGGGCCTCTAGCTGC | GCTCTGAGTGAAGCCA |
