# Supplementary material for: BRCA1: A Novel Prognostic Factor in Resected Non-Small-Cell Lung Cancer
Source: PLoS One. 2007 Nov 7;2(11):e1129. doi: 10.1371/journal.pone.0001129 (PMC2042516; doi:10.1371/journal.pone.0001129)
Supplement: Table S3 — Relative gene expression values (0.03 MB DOC) [file pone.0001129.s008.doc]

|  | Mean±sd | Median (range) |
| --- | --- | --- |
| ERCC1 | 1.50±1.03 | 1.23 (0.23-7.34) |
| MZF1 | 0.71±0.82 | 0.53 (0.03-6.72) |
| Twist | 13.23±15.29 | 7.75 (0.14-76.01) |
| RRM1 | 2.10±1.34 | 1.65 (0.37-6.86) |
| TRX | 2.71±2.45 | 1.82 (0.31-11.88) |
| Tdp1 | 1.77±0.98 | 1.57 (0.14-7.32) |
| NFAT | 0.56±0.39 | 0.46 (0.08-2.28) |
| BRCA1 | 4.33±3.54 | 3.65 (0.09-17.48) |
| BubR1 | 16.40±15.4 | 12.30 (0.85-90.06) |
