# Supplementary material for: BRCA1: A Novel Prognostic Factor in Resected Non-Small-Cell Lung Cancer
Source: PLoS One. 2007 Nov 7;2(11):e1129. doi: 10.1371/journal.pone.0001129 (PMC2042516; doi:10.1371/journal.pone.0001129)
Supplement: Table S4 — Gene expression according to disease stage (0.04 MB DOC) [file pone.0001129.s009.doc]

|  | Stage I | Stage II | Stage III | P* |
| --- | --- | --- | --- | --- |
|  | Median (range) | Median (range) | Median (range) |  |
| ERCC1 | 1.3 (0.23-7.34) | 1.2 (0.3-4.5) | 1.1 (0.3-4.7) | 0.94 |
| MZF1 | 0.48 (0.04-3.81) | 0.6 (0.04-6.7) | 0.5 (0.03-1.6) | 0.52 |
| Twist | 5.44 (0.17-69) | 9.5 (0.1-76) | 9.9 (0.2-53.2) | 0.14 |
| RRM1 | 1.6 (0.6-6.9) | 1.9 (0.6-4.7) | 1.5 (0.4-5.7) | 0.54 |
| TRX | 2 (0.31-11.88) | 1.5 (0.5-7.3) | 1.8 (0.4-7.2) | 0.72 |
| Tdp1 | 1.6 (0.7-7.3) | 1.7 (0.3-5) | 1.3 (0.1-4.2) | 0.23 |
| NFAT | 0.5 (0.1-2.3) | 0.4 (0.1-0.9) | 0.5 (0.1-2.2) | 0.49 |
| BRCA1 | 3.3 (0.3-17.2) | 4.1 (0.1-18.5) | 3.5 (0.2-6.9) | 0.41 |
| BubR1 | 11.5 (0.8-90) | 16.3 (3.4-83.3) | 14.2 (1.3-70.7) | 0.17 |

*Kruskal-Wallis
