# Supplementary material for: BRCA1: A Novel Prognostic Factor in Resected Non-Small-Cell Lung Cancer
Source: PLoS One. 2007 Nov 7;2(11):e1129. doi: 10.1371/journal.pone.0001129 (PMC2042516; doi:10.1371/journal.pone.0001129)
Supplement: Table S5 — Event-free survival according to gene expression levels (0.06 MB DOC) [file pone.0001129.s010.doc]

|  | N* | Event-free survival  (months) | 95% CI | P |
| --- | --- | --- | --- | --- |
| ERCC1 |  |  |  | 0.89 |
| ≤1.24 | 56 | NR | - |  |
| >1.24 | 57 | 38 | - |  |
| MZF1 |  |  |  | 0.29 |
| ≤0.43 | 55 | NR | - |  |
| >0.43 | 56 | 26 | - |  |
| Twist |  |  |  | 0.85 |
| ≤7.75 | 57 | 38 | - |  |
| >7.75 | 55 | NR | - |  |
| RRM1 |  |  |  | 0.11 |
| ≤1.65 | 61 | NR | - |  |
| >1.65 | 60 | 25 | 14.4-35.6 |  |
| TRX |  |  |  | 0.02 |
| ≤0.8 | 21 | NR | - |  |
| >0.8 | 93 | 32 | - |  |
| Tdp1 |  |  |  | 0.86 |
| ≤1.57 | 60 | NR | - |  |
| >1.57 | 61 | 35 | - |  |
| NFAT |  |  |  | 0.29 |
| ≤0.46 | 61 | NR | - |  |
| >0.46 | 61 | 35 | 18.4-51.5 |  |
| BRCA1 |  |  |  | 0.04 |
| ≤5 | 77 | NR | - |  |
| >5 | 36 | 22 | 14.9-29 |  |
| BubR1 |  |  |  | 0.44 |
| ≤12.28 | 61 | NR | - |  |
| >12.28 | 61 | 31 | - |  |

NR=not reached

*Event-free survival data is not available for some patients. Gene amplification was not successfully performed in all samples for all genes.
