# Supplementary material for: BRCA1: A Novel Prognostic Factor in Resected Non-Small-Cell Lung Cancer
Source: PLoS One. 2007 Nov 7;2(11):e1129. doi: 10.1371/journal.pone.0001129 (PMC2042516; doi:10.1371/journal.pone.0001129)
Supplement: Table S6 — Event-free survival in stage I patients according to gene expression levels (0.06 MB DOC) [file pone.0001129.s011.doc]

|  | N* | Event-free survival (months) | 95% CI | P |
| --- | --- | --- | --- | --- |
| ERCC1 |  |  |  | 0.09 |
| ≤1.5 | 41 | NR | - |  |
| >1.5 | 22 | 31 | 16.1-45.9 |  |
| MZF1 |  |  |  | 0.003 |
| ≤0.5 | 31 | NR | - |  |
| >0.5 | 29 | 26 | 12.7-39.3 |  |
| Twist |  |  |  | 0.54 |
| ≤9 | 38 | NR | - |  |
| >9 | 23 | NR | - |  |
| RRM1 |  |  |  | 0.11 |
| ≤1.63 | 35 | NR | - |  |
| >1.63 | 35 | NR | - |  |
| TRX |  |  |  | 0.11 |
| ≤0.8 | 15 | NR | - |  |
| >0.8 | 56 | NR | - |  |
| Tdp1 |  |  |  | 0.27 |
| ≤1.6 | 35 | NR | - |  |
| >1.6 | 36 | 38 | - |  |
| NFAT |  |  |  | 0.35 |
| ≤0.46 | 35 | NR | - |  |
| >0.46 | 36 | NR | - |  |
| BRCA1 |  |  |  | 0.04 |
| ≤5 | 43 | NR | - |  |
| >5 | 19 | 26 | 15.2-36.7 |  |
| BubR1 |  |  |  | 0.93 |
| ≤11.31 | 35 | NR | - |  |
| >11.31 | 36 | NR | - |  |

NR=not reached

*Event-free survival data is not available for some patients. Gene amplification was not successfully performed in all samples for all genes.
