# Supplementary material for: BRCA1: A Novel Prognostic Factor in Resected Non-Small-Cell Lung Cancer
Source: PLoS One. 2007 Nov 7;2(11):e1129. doi: 10.1371/journal.pone.0001129 (PMC2042516; doi:10.1371/journal.pone.0001129)
Supplement: Table S7 — Median survival for stage I patients according to gene expression levels (0.06 MB DOC) [file pone.0001129.s012.doc]

|  | N* | Median Survival (months) | 95% CI | P |
| --- | --- | --- | --- | --- |
| ERCC1 |  |  |  | 0.003 |
| ≤1.5 | 47 | NR | - |  |
| >1.5 | 24 | 29 | 19.9-38.1 |  |
| MZF1 |  |  |  | 0.002 |
| ≤0.5 | 36 | NR | - |  |
| >0.5 | 32 | 33 | 22.9-43 |  |
| Twist |  |  |  | 0.04 |
| ≤9 | 42 | NR | - |  |
| >9 | 27 | 29 | - |  |
| RRM1 |  |  |  | 0.06 |
| ≤1.63 | 35 | NR | - |  |
| >1.63 | 35 | 36.7 | 24.8-48.6 |  |
| TRX |  |  |  | 0.11 |
| ≤0.8 | 15 | NR | - |  |
| >0.8 | 56 | NR | - |  |
| Tdp1 |  |  |  | 0.49 |
| ≤1.6 | 35 | NR | - |  |
| >1.6 | 36 | 41.3 | 33-49.6 |  |
| NFAT |  |  |  | 0.74 |
| ≤0.46 | 35 | 41.3 | - |  |
| >0.46 | 36 | NR | - |  |
| BRCA1 |  |  |  | 0.02 |
| ≤5 | 48 | NR | - |  |
| >5 | 21 | 29 | 23.6-34.4 |  |
| BubR1 |  |  |  | 0.31 |
| ≤11.31 | 35 | NR | - |  |
| >11.31 | 36 | 39.5 | 31-48 |  |

NR=not reached

*Survival data is not available for some patients. Gene amplification was not successfully performed in all samples for all genes.
