# Supplementary material for: BRCA1: A Novel Prognostic Factor in Resected Non-Small-Cell Lung Cancer
Source: PLoS One. 2007 Nov 7;2(11):e1129. doi: 10.1371/journal.pone.0001129 (PMC2042516; doi:10.1371/journal.pone.0001129)
Supplement: Table S8 — Multivariate Cox model for survival, showing a greater risk of death for patients with high levels of BRCA1 and for those with stage IIIA disease (0.03 MB DOC) [file pone.0001129.s013.doc]

|  | Hazard Ratio | 95% C.I. | P |
| --- | --- | --- | --- |
| BRCA1 |  |  |  |
| ≤5 | 1 |  |  |
| >5 | 1.98 | 1.11-6 | 0.02 |
| Disease Stage |  |  |  |
| IA | 1 |  |  |
| IB | 2.41 | 0.71-8.20 | 0.16 |
| IIB | 2.21 | 0.62-7.89 | 0.22 |
| IIIA | 7.91 | 2.27-27.54 | 0.001 |
