# Supplementary material for: BRCA1: A Novel Prognostic Factor in Resected Non-Small-Cell Lung Cancer
Source: PLoS One. 2007 Nov 7;2(11):e1129. doi: 10.1371/journal.pone.0001129 (PMC2042516; doi:10.1371/journal.pone.0001129)
Supplement: Text S1 — Further details on the nine genes examined (0.09 MB DOC) [file pone.0001129.s014.doc]

We examined the following nine genes: excision repair cross-complementing 1 (ERCC1), BRCA1, human homolog of yeast budding uninhibited by benzimidazole (BubR1), myeloid zinc finger 1 (MZF1), Twist, ribonucleotide reductase subunit M1 (RRM1), thioredoxin-1 (TRX1), tyrosyl-DNA phosphodiesterase (Tdp1), and nuclear factor of activated T cells (NFAT).

Recently, the protein expression of ERCC1[1] and RRM1[2] has been associated with outcome in resected NSCLC; however, in a prior study in gastric cancer[3], it was unclear whether the poor clinical response of patients whose tumors had high pretreatment mRNA levels of ERCC1 resulted from tumor cell resistance to cisplatin-based chemotherapy or from a more aggressive tumor biology.

ERCC1 belongs to the global NER pathway of DNA repair[4], while BRCA1 is involved in transcription-coupled NER[5]. BRCA1 was overexpressed in cisplatin-resistant MCF-7 cells[6]. In contrast, BRCA1 induced increased sensitivity to paclitaxel and vinorelbine[7]. In neoadjuvant gemcitabine/cisplatin-treated stage II-IIIA NSCLC patients, the longest survival was observed in patients whose tumors had the lowest BRCA1 mRNA expression, while the shortest survival was observed in those with the highest expression[8]. The response rate in BRCA1-positive breast cancer patients was also significantly lower following neoadjuvant treatment with docetaxel than with DNA-damaging therapies[9].

BRCA1 regulates the expression of a number of genes involved in the spindle assembly checkpoint, includingBubR1[10]. BRCA1 upregulates BubR1 transcription, and BubR1 transcription and expression are significantly downregulated in MCF-7 cells with low levels of BRCA1[11]. Colorectal cancer patients with high BubR1 expression had significantly shorter relapse-free survival than those with normal expression[12].

MZF1 is a transcription factor of the Kruppel family of zinc finger proteins originally isolated from the peripheral blood leukocytes of a patient with chronic myeloid leukemia[13]. It is suggested that MZF1 could be a repressor of ERCC1 transcription upon cisplatin exposure[14]. Intriguingly, MZF1 overexpression induces N-cadherin promoter activity in osteoblasts[15]. N-cadherin molecules are expressed during metastatic progression. Twist, which plays an essential role in metastasis[16], is a pivotal transcription factor that upregulates the gene expression of N-cadherin during cancer metastasis[17].

RRM1 is required for DNA synthesis and repair . Patients in the lowest quartile of RRM1 mRNA expression had significantly longer survival following neoadjuvant gemcitabine/cisplatin chemotherapy in stage II-IIIA NSCLC[18]. TRX1 functions as a hydrogen donor for many protein targets and is required for ribonucleotide reductase activity[19]. Proteomic analysis identified TRX1 as overexpressed in NSCLC and correlated with poor survival[20]. TRX1 overexpression activates hypoxia-inducible factor 1 (HIF-1)[21] and is associated with lymph node metastasis and poor outcome in NSCLC[22]. Tdp1 may function in collaboration with multiple pathways involved in strand break repair[23,24]. Tdp1 expression is increased in the majority of NSCLCs, by Western blot[25]. Nuclear factor of activated T cells (NFAT) is observed in breast cancer cell lines and tissue[26]; active NFAT promotes carcinoma invasion *in vitro*[27].

1. Olaussen KA, Dunant A, Fouret P, Brambilla E, Andre F, et al. (2006) DNA repair by ERCC1 in non-small-cell lung cancer and cisplatin-based adjuvant chemotherapy. N Engl J Med 355: 983-991.

2. Zheng Z, Chen T, Li X, Haura E, Sharma A, et al. (2007) DNA synthesis and repair genes RRM1 and ERCC1 in lung cancer. N Engl J Med 356: 800-808.

3. Metzger R, Leichman CG, Danenberg KD, Danenberg PV, Lenz HJ, et al. (1998) ERCC1 mRNA levels complement thymidylate synthase mRNA levels in predicting response and survival for gastric cancer patients receiving combination cisplatin and fluorouracil chemotherapy. J Clin Oncol 16: 309-316.

4. Niedernhofer LJ, Odijk H, Budzowska M, van Drunen E, Maas A, et al. (2004) The structure-specific endonuclease Ercc1-Xpf is required to resolve DNA interstrand cross-link-induced double-strand breaks. Mol Cell Biol 24: 5776-5787.

5. Horwitz AA, Affar el B, Heine GF, Shi Y, Parvin JD (2007) A mechanism for transcriptional repression dependent on the BRCA1 E3 ubiquitin ligase. Proc Natl Acad Sci U S A 104: 6614-6619.

6. Husain A, He G, Venkatraman ES, Spriggs DR (1998) BRCA1 up-regulation is associated with repair-mediated resistance to cis-diamminedichloroplatinum(II). Cancer Res 58: 1120-1123.

7. Quinn JE, Kennedy RD, Mullan PB, Gilmore PM, Carty M, et al. (2003) BRCA1 functions as a differential modulator of chemotherapy-induced apoptosis. Cancer Res 63: 6221-6228.

8. Taron M, Rosell R, Felip E, Mendez P, Souglakos J, et al. (2004) BRCA1 mRNA expression levels as an indicator of chemoresistance in lung cancer. Hum Mol Genet 13: 2443-2449.

9. Byrski T, Gronwald J, Huzarski T, Grzybowska E, Budryk M, et al. (2007) Response to neo-adjuvant chemotherapy in women with BRCA1-positive breast cancers. Breast Cancer Res Treat.

10. Bae I, Rih JK, Kim HJ, Kang HJ, Haddad B, et al. (2005) BRCA1 regulates gene expression for orderly mitotic progression. Cell Cycle 4: 1641-1666.

11. Chabalier C, Lamare C, Racca C, Privat M, Valette A, et al. (2006) BRCA1 downregulation leads to premature inactivation of spindle checkpoint and confers paclitaxel resistance. Cell Cycle 5: 1001-1007.

12. Shichiri M, Yoshinaga K, Hisatomi H, Sugihara K, Hirata Y (2002) Genetic and epigenetic inactivation of mitotic checkpoint genes hBUB1 and hBUBR1 and their relationship to survival. Cancer Res 62: 13-17.

13. Hromas R, Collins SJ, Hickstein D, Raskind W, Deaven LL, et al. (1991) A retinoic acid-responsive human zinc finger gene, MZF-1, preferentially expressed in myeloid cells. J Biol Chem 266: 14183-14187.

14. Yan QW, Reed E, Zhong XS, Thornton K, Guo Y, et al. (2006) MZF1 possesses a repressively regulatory function in ERCC1 expression. Biochem Pharmacol 71: 761-771.

15. Le Mee S, Fromigue O, Marie PJ (2005) Sp1/Sp3 and the myeloid zinc finger gene MZF1 regulate the human N-cadherin promoter in osteoblasts. Exp Cell Res 302: 129-142.

16. Yang J, Mani SA, Donaher JL, Ramaswamy S, Itzykson RA, et al. (2004) Twist, a master regulator of morphogenesis, plays an essential role in tumor metastasis. Cell 117: 927-939.

17. Alexander NR, Tran NL, Rekapally H, Summers CE, Glackin C, et al. (2006) N-cadherin gene expression in prostate carcinoma is modulated by integrin-dependent nuclear translocation of Twist1. Cancer Res 66: 3365-3369.

18. Rosell R, Felip E, Taron M, Majo J, Mendez P, et al. (2004) Gene expression as a predictive marker of outcome in stage IIB-IIIA-IIIB non-small cell lung cancer after induction gemcitabine-based chemotherapy followed by resectional surgery. Clin Cancer Res 10: 4215s-4219s.

19. Yoshida T, Nakamura H, Masutani H, Yodoi J (2005) The involvement of thioredoxin and thioredoxin binding protein-2 on cellular proliferation and aging process. Ann N Y Acad Sci 1055: 1-12.

20. Yanagisawa K, Shyr Y, Xu BJ, Massion PP, Larsen PH, et al. (2003) Proteomic patterns of tumour subsets in non-small-cell lung cancer. Lancet 362: 433-439.

21. Welsh SJ, Bellamy WT, Briehl MM, Powis G (2002) The redox protein thioredoxin-1 (Trx-1) increases hypoxia-inducible factor 1alpha protein expression: Trx-1 overexpression results in increased vascular endothelial growth factor production and enhanced tumor angiogenesis. Cancer Res 62: 5089-5095.

22. Kakolyris S, Giatromanolaki A, Koukourakis M, Powis G, Souglakos J, et al. (2001) Thioredoxin expression is associated with lymph node status and prognosis in early operable non-small cell lung cancer. Clin Cancer Res 7: 3087-3091.

23. Interthal H, Chen HJ, Kehl-Fie TE, Zotzmann J, Leppard JB, et al. (2005) SCAN1 mutant Tdp1 accumulates the enzyme--DNA intermediate and causes camptothecin hypersensitivity. Embo J 24: 2224-2233.

24. Barthelmes HU, Habermeyer M, Christensen MO, Mielke C, Interthal H, et al. (2004) TDP1 overexpression in human cells counteracts DNA damage mediated by topoisomerases I and II. J Biol Chem 279: 55618-55625.

25. Liu C, Zhou S, Begum S, Sidransky D, Westra WH, et al. (2007) Increased expression and activity of repair genes TDP1 and XPF in non-small cell lung cancer. Lung Cancer 55: 303-311.

26. Yoeli-Lerner M, Yiu GK, Rabinovitz I, Erhardt P, Jauliac S, et al. (2005) Akt blocks breast cancer cell motility and invasion through the transcription factor NFAT. Mol Cell 20: 539-550.

27. Jauliac S, Lopez-Rodriguez C, Shaw LM, Brown LF, Rao A, et al. (2002) The role of NFAT transcription factors in integrin-mediated carcinoma invasion. Nat Cell Biol 4: 540-544.
